# Comments on “H4K12 Lactylation‐Activated Spp1 in Reprogrammed Microglia Improves Functional Recovery After Spinal Cord Injury”

**DOI:** 10.1111/cns.70339

**Published:** 2025-03-12

**Authors:** Jieqi Zhang, Kelin He, Ruijie Ma

**Affiliations:** ^1^ Department of Neurobiology and Acupuncture Research, Key Laboratory of Acupuncture and Neurology of Zhejiang Province, The Third School of Clinical Medicine (School of Rehabilitation Medicine) Zhejiang Chinese Medical University Hangzhou China; ^2^ Department of Acupuncture The Third Affiliated Hospital of Zhejiang Chinese Medical University Hangzhou China

Dear Editor,

We read with great interest the recent study by Wang et al. [1] on the role of H4K12 lactylation (H4K12la) in promoting Spp1 transcription and facilitating functional recovery after spinal cord injury (SCI). The work provides novel insights into the relationship between metabolic reprogramming in microglia and neurorepair. By utilizing recombinant Spp1 protein (rSPP1), the authors have demonstrated its significant role in functional recovery, and we believe this study has important implications for spinal cord injury research.

We would like to offer a few suggestions that could further strengthen the study's reliability and completeness.

First, regarding the overexpression experiments, the authors used recombinant Spp1 protein (rSPP1) to investigate its effects on functional recovery following SCI. It is crucial to confirm the successful overexpression of Spp1 protein. Given that the half‐life of recombinant proteins may vary depending on their properties and the administration route, confirming the protein expression levels at different time points will be essential for understanding its biological effects [2, 3]. We recommend the inclusion of Western blot (WB) results for both the vector group and recombinant Spp1 protein group at multiple time points to verify the expression levels of Spp1, particularly to confirm the duration of stable overexpression. This will help assess whether the overexpression effect of Spp1 is maintained during critical time windows of the experiment, which will strengthen the reliability of the observed functional recovery results.

Additionally, while the authors conducted separate experiments with neurons and microglia, there was no mention of coculture experiments involving both cell types. We suggest the authors include coculture experiments to investigate the bidirectional interaction between Spp1 in neurons and microglia, particularly how Spp1 may promote neuronal recovery by modulating the activation of microglia. This would offer a more comprehensive understanding of the mechanism by which Spp1 contributes to SCI repair.

Overall, we commend the authors for their innovative work and believe that their findings significantly advance our understanding of Spp1 in spinal cord injury repair. We look forward to further exploration of Spp1 and its regulatory mechanisms in this context.

Thank you for considering our suggestions, and we anticipate the continued development and refinement of this important work.

## Conflicts of Interest

The authors declare no conflicts of interest.

## Data Availability

Data sharing is not applicable to this article as no datasets were generated or analyzed during the current study.
